# Efficacy of mometasone furoate for nasal polyps: A protocol for systematic review of randomized controlled trial

**DOI:** 10.1097/MD.0000000000016632

**Published:** 2019-07-26

**Authors:** Hai-jiang Yu, Lin Han, Wei-feng Wang, Lin-hong Yang, Yu-fei Nie

**Affiliations:** a Department of Otorhinolaryngology, First Affiliated Hospital of Jiamusi University; b Department of Ear-Nose-Throat, The 963rd Hospital of the Joint Logistic Support Force of the Chinese People's Liberation Army; c Department of Neurosurgery, First Affiliated Hospital of Jiamusi University; d Department of Otorhinolaryngology, People's Hospital of Jiansanjiang, Jiamusi, China.

**Keywords:** efficacy, mometasone furoate, nasal polyps, randomized controlled trial, safety

## Abstract

**Background:**

This study aims to systematically explore the efficacy and safety of mometasone furoate (MTF) for patients with nasal polyps (NP).

**Methods:**

We will search MEDLINE, Cochrane Library, PubMed, Springer, Web of Science, Ovid, Wangfang and Chinese Biomedical Literature Database from their inception to April 30, 2019 without language restrictions. All randomized controlled trials (RCTs) of MTF for the treatment of NP will be considered for inclusion. RevMan 5.3 software will be used for data synthesis, subgroup analysis, sensitivity analysis, as well as the meta-analysis.

**Results:**

Primary outcomes include change in symptom scores (as measured by any symptom scores), and polyp size (as assessed by any Polyp size scores or tools). Secondary outcomes consist of polyp recurrence, change in nasal air flow, quality of life outcomes (as measured by any quality of life scales, such as Short Form Health Survey is a 36-item), and adverse events.

**Conclusion:**

This study will provide evidence for judging whether MTF is an effective and safe treatment for NP or not.

**PROSPERO registration number:**

PROSPERO CRD42019134037.

## 1 Introduction

Nasal polyp (NP) is benign growths of the mucosa.^[1–4]^ It is manifested that this disorder includes nasal obstruction, nasal discharge, postnasal drip, and loss of smell.^[5–7]^ If such disorder cannot treat fairly well, it can cause recurrent nature of NP and can significant impact heath-related quality of life.^[8–10]^ It has been estimated that its prevalence is up to 4%, which indicating a significant medical need for effective treatment.^[11–13]^

A variety of clinical studies have reported that mometasone furoate (MTF) can be utilized to treat NP effectively.^[14–24]^ However, no study has systematically assessed the efficacy and safety of MTF for the treatment of patients with NP. Therefore, in this study, we will systematically explore the efficacy and safety of MTF for patients with NP.

## 2 Methods and materials

### 2.1 Ethics and dissemination

This study does not need ethic approval because no individual data will be used. Its results are expected to be published in peer-reviewed journals.

### 2.2 Study registration

This study has been registered as PROSPERO CRD42019134037. Its reports follow the guideline of Preferred Reporting Items for Systematic Reviews and Meta-Analysis (PRISRMA) Protocol statement.^[25]^

### 2.3 Eligibility criteria for study selection

#### 2.3.1 Type of studies

Only randomized controlled trials (RCTs) on assessing the efficacy and safety of MTF for patients with NP will be included regardless language restrictions. Non-clinical studies, non-controlled trials, and non-RCTs will be excluded.

#### 2.3.2 Type of participants

Patients diagnosed as NP will be considered regardless their age, gender, ethnicity, and severity of NP.

#### 2.3.3 Type of interventions

The experimental group must have been treated with MTF monotherapy.

The control group can be treated with any interventions, except any forms of MTF.

#### 2.3.4 Types of outcomes

##### 2.3.4.1 Primary outcomes

Change in symptom scores (as measured by any symptom scores);

Polyp size (as assessed by any Polyp size scores or tools);

##### 2.3.4.2 Secondary outcomes

Polyp recurrence;

Change in nasal air flow;

Quality of life outcomes (as measured by any quality of life scales, such as Short Form Health Survey is a 36-item);

Adverse effects (any expected and unexpected adverse events).

### 2.4 Search methods for the identification of studies

#### 2.4.1 Search strategy

Relevant RCTs of MTF for NP from the following databases of Cochrane Library, MEDLINE, PubMed, Springer, Web of Science, Ovid, Wangfang and Chinese Biomedical Literature Database will be searched from their inception to April 30, 2019 with language restrictions. The strategy for searching the Cochrane Library will be presented as an example in Table 1. The modified search strategy will also be applied to other electronic databases.

**Table 1:**
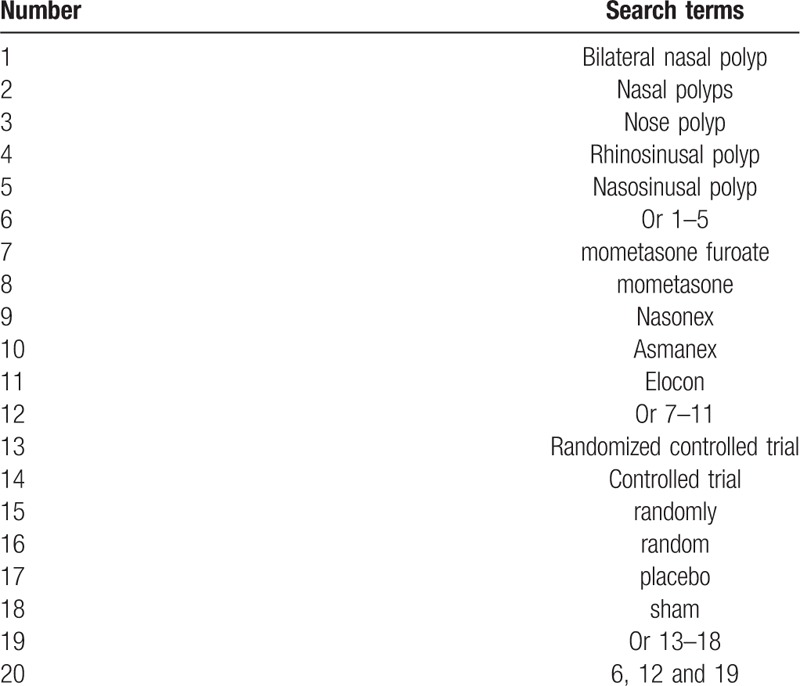
Search strategy applied in MEDLINE database.

Additionally, gray literatures including dissertations, relevant conference proceedings, and reference lists of associated reviews will also be identified to avoid missing any potential eligible studies.

#### 2.4.2 Dealing with insufficient information

Whenever the information is insufficient or missing, primary authors will be contacted to inquire those data. If we cannot receive that information, the available data will be analyzed only.

#### 2.4.3 Study selection

Two authors will independently carry out study selection by reading the title and abstracts of all potential studies and then eliminating the duplicated literature and irrelevant articles in accordance with the previous eligibility criteria. Then, full texts will be read to further determine whether they will finally meet all included criteria or not. When there are disagreements, they will be solved by discussion with another author. The process of all study selection is shown in PRISMA flow diagram.

#### 2.4.4 Data extraction

Two authors will carry out data extraction from each eligible literature according to the pre-diluted data extraction sheet. The sheet comprise of the following information: literature title, authors, date of publication, sample size, patient characteristics, study methods, treatment details, outcome indicators, as well as other factors for risk of bias assessment. Any divergences between 2 authors will be solved by discussion with a third author.

#### 2.4.5 Methodology quality assessment

Cochrane risk of bias tool will be used for methodological quality assessment for each eligible study. It includes random sequence, allocation concealment, blinding of participants and patients, blinding of outcome measurements, incomplete outcomes, selective publication, and other risk of bias. Each of them will be further classified as high, unclear, or low risk of bias. Two authors will judge the methodology quality for all eligible studies. When there is inconsistency, a third author will be consulted to solve the disagreements.

### 2.5 Data synthesis and analysis

#### 2.5.1 Measurement of treatment effect

For continuous data, they will be shown as weighted mean difference or standardized mean difference with 95% confidence intervals (CIs). For dichotomous data, they will be shown as risk ratio or odds ratio with 95% CIs.

#### 2.5.2 Assessment of heterogeneity

Heterogeneity will be determined by *I*^2^ test. If *I*^2^ ≤ 50%, heterogeneity is considered as minor. If *I*^2^ > 50%, heterogeneity is considered as substantial, and subgroup analysis will be conducted to identify any possible sources of heterogeneity.

#### 2.5.3 Data synthesis

If heterogeneity is minor, a fixed-effect model will be used for data pooling, and meta-analysis will be carried out. If heterogeneity is substantial, random-effect model will be used for data pooling, and meta-analysis will be carried out according to the results of subgroup analysis. If there is minor heterogeneity after subgroup analysis, meta-analysis will be performed. Otherwise, we will not pool the data and meta-analysis will not be operated. Instead, we will only perform narrative summary reports.

#### 2.5.4 Subgroup analysis

Subgroup analysis will be conducted to check any possible factors causing significant heterogeneity based on the different treatments, controls, and outcome measurements.

#### 2.5.5 Sensitivity analysis

Sensitivity analysis will be adopted to determine the robustness and stability of pooled outcome data by removing low methodological quality studies.

#### 2.5.6 Publication bias

Funnel plot^[26]^ and Egger regression test^[27]^ will be carried out to check any reporting bias if more than 10 eligible studies is included.

## 3 Discussion

The protocol of this study will summarize the latest data to evaluate the efficacy and safety of MTF for patients with NP. The results of this study will provide the helpful evidence whether MTF can exert efficacy and acceptable safety of MTF for the treatment of patients with NP. Additionally, the findings of this study may also provide a useful reference for implementation of MTF and collection of clinical data for practitioners, researchers, and health policy makers.

## Author contributions

**Conceptualization:** Hai-jiang Yu, Lin Han, Wei-feng Wang, Lin-hong Yang, Yu-fei Nie.

**Data curation:** Hai-jiang Yu, Lin Han, Wei-feng Wang, Lin-hong Yang, Yu-fei Nie.

**Formal analysis:** Hai-jiang Yu, Wei-feng Wang, Lin-hong Yang.

**Funding acquisition:** Yu-fei Nie.

**Investigation:** Lin-hong Yang, Yu-fei Nie.

**Methodology:** Hai-jiang Yu, Lin Han, Wei-feng Wang.

**Project administration:** Yu-fei Nie.

**Resources:** Hai-jiang Yu, Lin Han, Wei-feng Wang, Lin-hong Yang.

**Software:** Lin Han, Lin-hong Yang.

**Supervision:** Lin Han, Wei-feng Wang, Yu-fei Nie.

**Validation:** Hai-jiang Yu, Wei-feng Wang, Lin-hong Yang, Yu-fei Nie.

**Visualization:** Hai-jiang Yu, Lin Han, Wei-feng Wang, Yu-fei Nie.

**Writing – original draft:** Hai-jiang Yu, Lin Han, Lin-hong Yang, Yu-fei Nie.

**Writing – review & editing:** Hai-jiang Yu, Wei-feng Wang, Lin-hong Yang, Yu-fei Nie.
